# Application of the Benchmark Dose (BMD) Method to Identify Thresholds of Cadmium-Induced Renal Effects in Non-Polluted Areas in China

**DOI:** 10.1371/journal.pone.0161240

**Published:** 2016-08-18

**Authors:** Xiaofeng Wang, Yu Wang, Lingfang Feng, Yan Tong, Zhijian Chen, Shibo Ying, Tianhui Chen, Tao Li, Hailing Xia, Zhaoqiang Jiang, Qi Shang, Xiaoming Lou, Jianlin Lou

**Affiliations:** 1 Department of Environmental and Occupational Health, Zhejiang Provincial Center for Disease Control and Prevention, Hangzhou 310051, Zhejiang, P.R. China; 2 Institute of Occupational Diseases, Zhejiang Academy of Medical Sciences, Hangzhou 310013, Zhejiang, P.R. China; 3 Institute for Environmental Health and Related Product Safety, Chinese Center for Disease Control and Prevention, Beijing 100050, P.R. China; Gentofte Hospital, DENMARK

## Abstract

The benchmark dose (BMD) method has been increasingly used to assess the health risks of cadmium (Cd) in epidemiological studies. The aim of our study was to estimate the threshold levels of urinary Cd (UCd) using the BMD method in a general population of Jiangshan City, Zhejiang Province of China. In our study, a total of 934 people (469 men, 465 women) were recruited and morning urine samples were collected from all the participants. Levels of Cd, creatinine, and renal dysfunction indicators such as retinol binding protein (RBP), *β*2-microglobulin (*β*2-MG), and N-acetyl-*b*-glucosaminidase (NAG) in urine were detected for analysis of BMD and BMD low (BMDL) of UCd. RBP, *β2*-MG, and NAG in urine all correlated significantly (*P* < 0.001) with UCd except of age (*P* = 0.767). When the benchmark response (BMR) was 5%, the BMD/BMDL of UCd for RBP, *β*2-MG, and NAG was 1.69/ 0.89, 1.24/0.62, 0.85/0.49 μg/g Cr in men and 1.70/0.76, 1.35/0.64, 1.36/0.65 μg/g Cr in women, respectively. If the BMR was set at 10%, the BMD/BMDL of UCd for RBP, *β*2-MG, and NAG was 2.44/1.59, 2.09/1.30, 1.80/1.04 μg/g Cr in men and 2.43/1.53, 2.10/1.34, 2.31/1.37 μg/g Cr in women, respectively. Our results provided evidence for Cd-induced tubular effects in cadmium non-polluted areas in China. Both *β*2-MG and NAG were more sensitive than RBP in response to Cd exposure. But *β*2-MG was the most sensitive indicator in women, and NAG was the most sensitive one in men.

## Introduction

Adverse effects of cadmium (Cd) on the human kidney have been demonstrated in both occupational workers and general people [1,2,3,4]. For the general population, diet and smoking are the main source of Cd, and long-term Cd exposure at low levels may lead to Cd accumulation in kidneys because of its long biological half-life, inducing glomerular and tubular dysfunctions [5,6,7]. Epidemiologic studies suggest that cadmium is associated with chronic kidney disease [8]. The best known example of environmental cadmium exposure is Itai-Itai disease in Japan, related to dietary exposure to cadmium from the contaminated waters. In order to protect people from injury induced by Cd exposure, it is very important to establish the reference exposure of Cd below which the risk of adverse response is low. In China, the environments of more than 30 places are also contaminated with cadmium [9], and the residents in these areas are at risk of renal effects due to long-term exposure to low levels of Cd. Other environmental factors such as heavy metals (lead, and mercury), occupational solvents, as well as various infectious agents [8, 10, 11, 12] are also likely to have an effect on kidney. Identification of these preventable risk factors of kidney disease could contribute to protect people from renal injury. We aimed to evaluate the potential relationship between cadmium and nephrotoxicity biomarkers and to estimate the BMDs of urinary Cd for Cd-induced tubular effects in a cadmium non-polluted area in China.

The benchmark dose (BMD) method is increasingly used to assess the health risks of environmental contaminants [13,14,15]. BMD is defined as the exposure level that corresponds to a specific increase in the probability of an adverse response (benchmark response, BMR). The benchmark dose low (BMDL) is defined as the value corresponding to the lower 95% confidence intervals of the BMD [13], and can be used in risk assessment as a replacement for the no observed adverse effect level (NOAEL). Unlike NOAEL, BMD is not constrained to one experimental dose, but utilizes the information from the whole dose-response curve. Additionally, BMD method takes into account the shape of the dose–response relationship to a greater extent and depends on less sample size compared with NOEAL [16]. However, only in a few studies [9,17,18,19] has the BMD method been used to estimate the threshold levels of urinary Cd for Chinese populations with no anthropogenic exposure to cadmium until now. Furthermore, studies suggested that both BMD and BMDL values vary substantially depending on the populations studied, and such is the case even when all data are from non-exposed populations in a single nation [20,21]. The aim of our present study was to determine the BMDs of urinary Cd for Cd-induced tubular effects in a cadmium non-polluted area in China, using the widely used BMD method.

Urinary Cd is widely used to assess exposure or body burden of Cd in the general population [6]. It is well known that there is a negative association between diuresis and concentrations of biomarkers in urine, therefore adjustment for dilution based on urine creatinine concentration (U-Cr) is used to avoid false-positive associations [22,23]. Low-molecular weight protein, such as urinary concentrations of *β*2-microglobulin (*β*2-MG), and retinol-binding protein (RBP) are valid markers of the tubular reabsorption. The urinary Nacetyl-β-d-glucosaminidase (NAG), an enzyme localized in the lysosomes of the tubular cells, is a sensitive marker of leakage from damaged tubular cells. In the present study, urinary RBP, urinary *β*2-MG and urinary NAG were used as indicators of renal tubular dysfunction markers in Cd-non-polluted populations.

## Materials and Methods

### Study population

The present study was carried out in Jiangshan City, a cadmium non-polluted area in the Southwest of Zhejiang Province. In this area, agriculture occupies the dominant position and its natural environmental condition is free from kinds of industrial and mining pollution. A total of five towns within Jiangshan City were selected and three soil samples and six rice samples in each town were collected for analysis of cadmium contents. The mean (SD) cadmium content was 0.06 (0.03) mg/kg in rice and 0.13 (0.02) mg/kg in soil, both of which were lower than the national standards of 0.2 mg/kg [24] and 0.3 mg/kg [25], respectively. We initially carried out an investigation in each town to acquire some basic information, including the number of people, sex, and age of the inhabitants. To ensure the representativeness of sampling and accuracy of the experiments, we then chose the inhabitants according to sex and age by stratified sampling. A total of 934 (469 men, 465 women) inhabitants were randomly recruited in our study. All of the subjects were local residents who mainly engaged in farming for occupation and subsisted on locally grown crops. Our research was performed according to a protocol approved by the Ethics Committee of Zhejiang Provincial Center for Disease Control and Prevention, and the ethics committee specifically approved to study the threshold levels of UCd using the BMD method in a general population of Jiangshan City, Zhejiang Province of China. No specific permissions were required for the locations and activities involved in our study because no protected area of land or sea was included, and the activities included in our studies were all routine exams. The subjects were requested to provide their written informed consent to participate in this study, fill out a detailed questionnaire, and provide urine samples for biological measurements. Those with diabetes, renal disease, urinary system disease, or occupational exposure to cadmium were excluded from our study. The number of subjects examined according to sex and age is shown in Table 1.

**Table 1:** Age distribution of subjects enrolled in our study.

Age (years)	Male	Female
< = 10	60	54
11–20	96	94
21–30	53	55
31–40	49	47
41–50	54	57
51–60	56	55
61–70	48	55
71+	53	48
Total	469	465

### Collection of samples and analytical method

Specimens of morning urine were collected from all participants and kept frozen at -20°C until analysis. The samples were collected in 250 ml polyethylene bottles soaked in 3 mol/l nitric acid for 16 h and rinsed in deionized water. Cadmium in urine (UCd) was measured by means of inductively coupled plasma-mass spectrometry (ICP-MS) with an (NexION 300D) instrument. Briefly, urine specimens (2 ml) were diluted one-fifth to 10 ml with a HNO_3_ 1% solution containing ^115^In as internal standards. Analysis was determined with isotopes of ^111^Cd and the detection and quantification limits were 0.05 μg/l. The relative standard deviation (RSD) was 5.1%, and recovery was 104%. We selected the following parameters as indicators of renal dysfunction: RBP, *β*2-MG, and NAG in urine. Urinary *β*2-MG was measured with radioimmunoassay (RIA) method on a γ counter (GAMMA-C12, DEEP, USA) by the China Institute of Atomic Energy. Activity of urinary NAG was detected by colorimetric method with a spectrophotometer (Model 721). RBP was analyzed with Retinol Binding Protein assay kit (enzyme linked immunosorbent assay, ELISA) (Sunbiote Company, Shanghai, China). The tests for RBP, *β*2-MG, and NAG were run in duplicates, and intra-assay variation coefficient was < 10%. Creatinine was measured using a Creatinine Assay Kit according to the manufacturer’s instructions. The test was made in triplicate and intra-assay variation coefficient was < 10%. Finally, all urinary parameters were adjusted for creatinine in urine.

### Statistical analysis

Regression analysis and curve estimation was performed with Benchmark Dose Software (version 2.4) available from the US Environmental Protection Agency (EPA). The levels of the urinary cadmium were confirmed to fit a log-normal distribution. Accordingly, urinary Cd was presented as geometric means (GM). Pearson linear correlation analysis Multiple and stepwise multiple linear regression analysis were performed to determine the factors influencing the relationships among urinary Cd levels, age, and three biomarkers (RBP, *β*2-MG, and NAG). The level of significance was set at P < 0.05. Cut-off values for the indicators of renal tubular dysfunction were defined as the 95% or 90% upper limit values of the population. If the value was higher than the cut-off point, we defined the renal function as abnormal (positive). The cut-off values of each substance are shown in Table 2 for each expressed unit and gender. Abnormal value of indicators for renal tubular dysfunction was set at 10% or 5%. The BMD and BMDL values were then calculated by a log-logistic model defined as follows: *P* [response] = background + [1-background]/[1+ EXP (-intercept-slope × log (dose))].

**Table 2:** Cut-off values of URBP, U*β*_*2*_-MG, and UNAG.

Indexes	Unit	Sex	Cut-off value	
			90%	95%
RBP	μg/g Cr	Male	272.92	542.08
		Female	227.00	498.66
*β*_*2*_MG	mg/g Cr	Male	0.78	1.39
		Female	0.69	1.77
NAG	U/g Cr	Male	16.49	25.93
		Female	17.82	24.81

Cut-off values are defined as the 90% or 95% upper limit values, which are calculated from the 934 persons who participated in this study.

## Results

### Correlation and regression analysis

**Fig 1:**
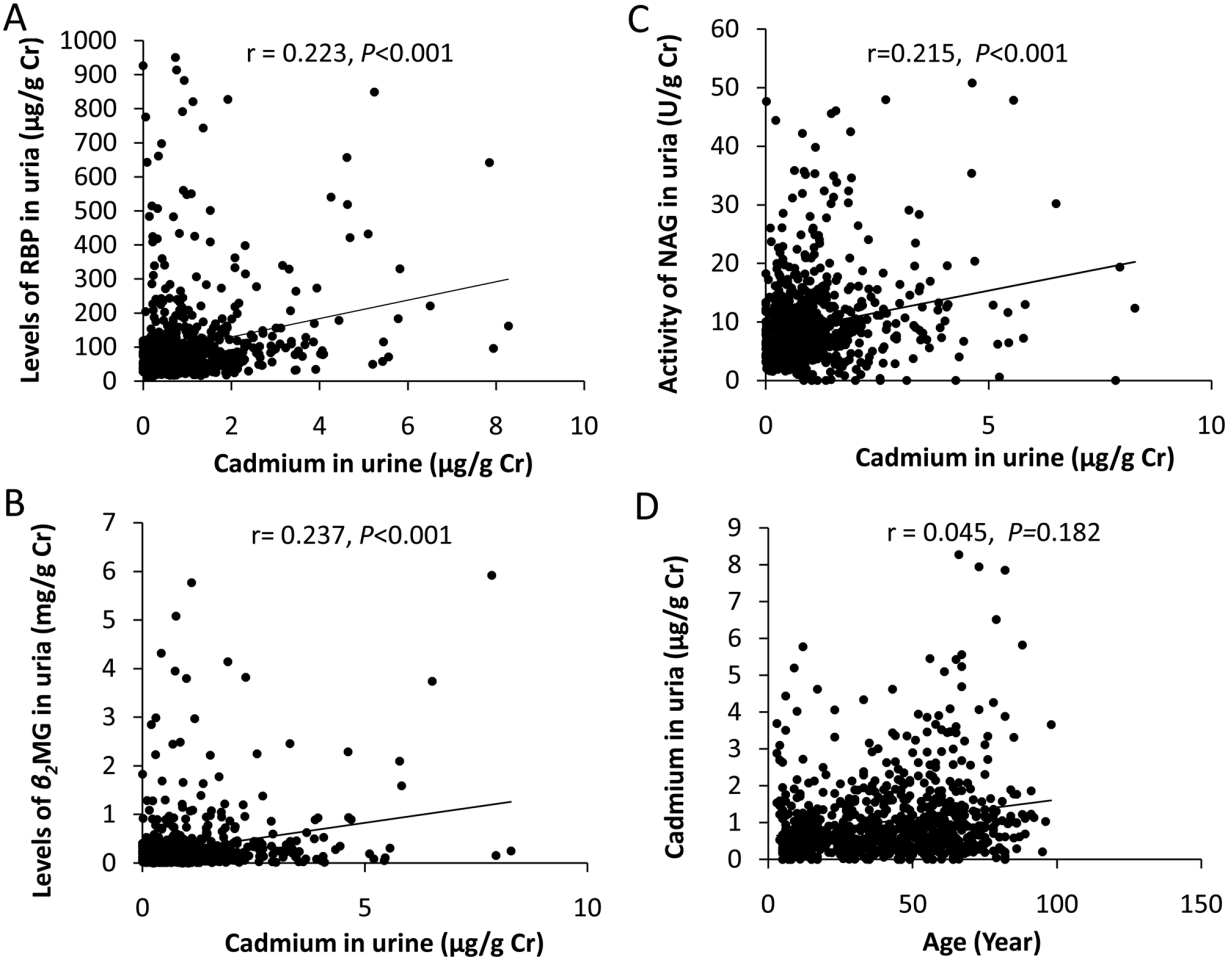
Correlations among age, three biomarkers (RBP, *β*2-MG, and NAG), and urinary Cd. r, correlation coefficient.

Fig 1 shows the relationships among age, three biomarkers (RBP, *β*2-MG, and NAG), and urinary Cd. All renal variables correlated significantly with urinary Cd in our study (Fig 1A–1C, *P* < 0.001) with the exception of age (Fig 1D, r = 0.045, *P* = 0.182). We conducted a stepwise multiple linear regression analysis with three biomarkers as dependent variables, and age and urinary Cd as independent variables. Our results showed that both age and urinary Cd correlated significantly (*P* < 0.001) with three biomarkers. The partial regression coefficients between three biomarkers and age were separately 1.172, 0.003, and 0.072 for URBP, Uβ2-MG, and UNAG, while those between the biomarkers and urinary Cd were 20.89, 0.118, and 1.143, individually. Urinary Cd showed to be a more influential variable compared with age.

### Prevalence of hyper-URBP (Table 3), hyper-Uβ2-MG (Table 4), and hyper-UNAG (Table 5) at different levels of urinary Cd by sex

At first, cut-off values for urinary substances were defined as corresponding to the 95% upper limit values of the target population, as those studies previously did by Kobayashi et al. [26] in a Japanese population and by Jin et al. [9] in a Chinese population. However, few subjects had levels over the thresholds. Therefore, the cut-off values were then defined as 90% upper limit values for all indicators. The subjects were divided into five groups according to UCd levels. That is, ≤0.49, 0.50–0.99, 1.00–1.49, 1.50–1.99, and ≥ 2.00 μg/g Cr, the geometric mean of UCd in each group was 0, 0.69, 1.20, 1.67, and 3.43 μg/g Cr, respectively. As shown in Tables 3–5, the prevalence of three indicators mostly increased with urinary Cd level in both males and females when 90% upper limit values were used. But this is not true with the prevalence of URBP if 95% upper limit values were employed. Thus, 90% was selected as the assumed cut-off point in our study.

**Table 3:** Prevalence of hyper-URBP at different levels of urinary Cd in males and females living in a Cd non-polluted area in China.

UCd (μg/g Cr)			Cut-off (90%)		Cut-off (95%)	
Range	GM	Total number	n	%	n	%
***Males***						
< = 0.49	0	159	14	8.8	5	3.1
0.50–0.99	0.69	127	5	3.9	5	3.9
1.00–1.49	1.20	62	9	14.5	7	11.3
1.50–1.99	1.67	24	3	12.5	2	8.3
2.00+	3.43	35	8	22.9	1	2.9
Total		407	39	9.6	20	4.9
Linear trend test			χ^2^ = 7.106, *P* = 0.008		χ^2^ = 1.335, *P* = 0.248	
***Females***						
< = 0.49	0	127	7	5.5	2	1.6
0.50–0.99	0.70	123	8	6.5	3	2.4
1.00–1.49	1.20	57	4	7	3	5.3
1.50–1.99	1.74	35	4	11.4	3	8.6
2.00+	3.53	59	14	23.7	8	13.6
Total		401	37	9.2	19	4.7
Linear trend test			χ^2^ = 14.718, *P*<0.001		χ^2^ = 14.547, *P*<0.001	

UCd, Urinary cadmium concentration; GM, geometric mean; n, number of subjects with hyper-URBP; %, prevalence of hyper-URBP. Cut-off (90%) or (95%), Cut-off values of URBP are 90% or 95% upper limit values calculated from 469 men and 465 women.

**Table 4:** Prevalence of hyper-U*β*_*2*_-MG at different levels of urinary Cd in males and females living in a Cd non-polluted area in China.

UCD (μg/g Cr)		Total number of subjects	Cut-off (90%)		Cut-off (95%)	
Range	GM		N	%	N	%
**Males**						
< = 0.49	0	163	10	6.1	5	3.1
0.50–0.99	0.69	131	7	5.3	2	1.5
1.00–1.49	1.20	66	9	13.6	6	9.1
1.50–1.99	1.67	26	4	15.4	1	3.8
2.00+	3.43	38	8	21.1	4	10.5
Total		424	38	9.0	18	4.2
Linear trend test			χ^2^ = 12.060, *P* = 0.001		χ^2^ = 5.387, *P* = 0.02	
**Females**						
< = 0.49	0	133	6	4.5	1	0.8
0.50–0.99	0.70	130	9	6.9	4	3.1
1.00–1.49	1.20	60	3	5.0	2	3.3
1.50–1.99	1.74	38	6	15.8	3	7.9
2.00+	3.53	62	15	24.2	10	16.1
Total		423	39	9.2	20	4.7
Linear trend test			χ^2^ = 19.479, *P*<0.001		χ^2^ = 21.309, *P*<0.001	

UCD, Urinary cadmium concentration; GM, geometric mean; n, number of subjects with hyper-U*β*_*2*_-MG; %, prevalence of hyper-U*β*_*2*_-MG. Cut-off (90%) or (95%), Cut-off values of U*β*_*2*_-MG are 90% or 95% upper limit values calculated from 469 men and 465 women.

**Table 5:** Prevalence of hyper-UNAG at different levels of urinary Cd in males and females living in a Cd non-polluted area in China.

UCD (μg/g Cr)		Total number	Cut-off (90%)		Cut-off (95%)	
Range	GM		N	%	N	%
**Males**						
< = 0.49	0	164	9	5.5	2	1.2
0.50–0.99	0.69	133	11	8.3	5	3.8
1.00–1.49	1.20	68	13	19.1	6	8.8
1.50–1.99	1.67	26	4	15.4	3	11.5
2.00+	3.43	38	6	15.8	4	10.5
Total		429	43	10	20	4.7
Linear trend test			χ^2^ = 10.075, *P* = 0.002		χ^2^ = 11.822, *P* = 0.001	
**Females**						
< = 0.49	0	136	10	7.4	3	2.2
0.50–0.99	0.70	131	9	6.9	4	3.1
1.00–1.49	1.20	62	7	11.3	2	3.2
1.50–1.99	1.74	38	7	18.4	5	13.2
2.00+	3.53	62	13	21	7	11.3
Total		429	46	10.7	21	4.9
Linear trend test			χ^2^ = 11.241, *P* = 0.001		χ^2^ = 11.227, *P* = 0.001	

UCD, Urinary cadmium concentration; GM, geometric mean; n, number of subjects with hyper-UNAG; %, prevalence of hyper-UNAG. Cut-off (90%) or (95%), Cut-off values of UNAG are 90% or 95% upper limit values calculated from 469 men and 465 women.

### BMD and BMDL values of UCd based on URBP, Uβ2-MG, and UNAG

The BMD and BMDL values for 90% cut-off values were calculated and shown in Table 6, with an abnormal value of 10% or 5% being employed.

**Table 6:** BMDL estimates of urinary Cd for URBP, U*β*_*2*_-MG, and UNAG.

Indicators	Sex	Cut-off values (%)	AIC	Intercept	Slope	*P*	BMD_10_	BMDL_10_	BMD_05_	BMDL_05_
RBP	Males	90	255.32	-4.02	2.04	0.07	2.44	1.59	1.69	0.89
	Females	90	238.14	-4.06	2.10	0.95	2.43	1.53	1.70	0.76
*β*_*2*_-MG	Males	90	251.52	-3.26	1.44	0.31	2.09	1.30	1.24	0.62
	Females	90	247.49	-3.45	1.68	0.43	2.10	1.34	1.35	0.64
NAG	Males	90	275.70	-2.80	1.00	0.24	1.8	1.04	0.85	0.49
	Females	90	288.80	-3.37	1.40	0.46	2.31	1.37	1.36	0.65

BMD_10_, Excess risk at BMR of 0.10; BMD_5_, Excess risk at BMR of 0.05; Cut-off (90%), Cut-off values are 90% upper limit values calculated from 469 men and 465 women.

The BMD values of urinary Cd for RBP, *β*_*2*_-MG, and NAG were 2.44, 2.09, 1.80 μg/g Cr in men and 2.43, 2.10, 2.31 μg/g Cr in women, respectively, and BMDL values were 1.59, 1.30, 1.04 μg/g Cr in men and 1.53, 1.34, 1.37 μg/g Cr in women, when an abnormal value of 10% was employed. If the abnormal value was set at 5%, the BMD values of urinary Cd for RBP, *β*_*2*_-MG, and NAG were 1.69, 1.24, 0.85 μg/g Cr in men and 1.70, 1.35, 1.36 μg/g Cr in women, respectively, and BMDL values were 0.89, 0.62, 0.49 μg/g Cr in men and 0.76, 0.64, 0.65 μg/g Cr in women.

## Discussion

According to our research, when BMR was set at 5%, the BMD/BMDL values of UCd for RBP, U*β*_*2*_-MG, and UNAG in Cd non-polluted areas in Zhejiang province of China ranged from 0.85/0.49 to 1.69/0.89 μg/g Cr in men and 1.36/0.64 to 1.70/0.76 μg/g Cr in women. When BMR was set at 10%, the BMD/BMDL values of UCd ranged from 1.8/1.04 to 2.44/1.59 μg/g Cr in men and 2.10/1.34 to 2.43/1.53 μg/g Cr in women. Similar results were reported in previous studies conducted in Japan or in China, although the values of BMD/BMDL varied among different studies. Kobayashi et al. [26,27] and Shimizuet al. [28] estimated the BMD/BMDL of inhabitants aged 50 years or more in cadmium non-polluted areas. Their results indicated that BMDL ranged from 2.0 to 4.0 μg/g Cr in men and 1.5 to 3.6 μg/g Cr in women, when cut-off values were 84% or 97.5% of urinary *β*_*2*_-MG or NAG. BMDL values estimated in our research were much lower than those in Japan. However, our results were approximate to another study conducted in China [18], and their results indicated that the estimated BMDL ranged from 0.88 to 1.24 μg/g Cr, when the cut-off values were defined as the upper 95% limit value and the BMR was set at 10%. Another study conducted in two cadmium polluted areas in China showed that the BMD/BMDL values of UCd for 35–55 year-old women were 1.07/0.44 and 2.12/0.53 μg/g Cr based on U*β*_*2*_-MG and UNAG, respectively, when a cut-off value of 90% and a BMR of 5% was set. Interestingly, the results of these studies, in which subjects from cadmium non-polluted areas in China were recruited [29], were somewhat consistent with our results. Previous studies suggested that even the analysis was conducted in a single nation, both BMD and BMDL for the Cd effect markers varied by fourfold (a1-MG or *β*_*2*_-MG) to sevenfold (NAG) changes among Cd-non-exposed populations [26,27]. Therefore, the estimation of BMD/BMDL values varied with populations, tubular indicators, cut-off values, or BMR being used. It has been known that the Benchmark Dose Software requires categorization of the subjects into several groups according to the magnitude of cadmium exposure. Categorization of the exposures could result in difference in the dose-intervals and the number of categories. These effects might partially explain the fluctuation on BMD/BMDL values estimated in different studies [21].

Based on published literature, the indicators most widely used for estimation of BMD/BMDL values of UCd were NAG and *β*_*2*_-MG, while RBP was not used as commonly as NAG or *β*_*2*_-MG. Low-molecular weight proteins (RBP and β2-MG) are produced at different extra-renal sites, freely filtered by the glomerulus, and reabsorbed but not secreted by proximal tubular cells [30,31]. Injury to the proximal tubular epithelium could lead to reduced re-absorptive capacity and the resultant tubular proteinuria. Increased urinaryβ2-MG excretion has been observed to be an early marker of tubular injury. N-acetyl-b-glucosaminidase (NAG) is a proximal tubule lysosomal enzyme and is among the best characterized urinary enzymes [32]. It has been suggested that the isoenzyme NAG-B exhibits a specific association with very low levels of urinary cadmium [33]. Consistently, our results also suggested that RBP was not as sensitive as NAG or *β*_*2*_-MG in estimating thresholds of cadmium-induced renal effects in cadmium non-polluted areas in China. The thresholds of urinary Cd levels were different when different dysfunction markers were used. Benchmark dose (BMD) studies on Cd-exposed populations showed a lower BMD for NAG than that for *β*_*2*_-MG [7, 14], whereas Kobayashi et al. [26,27] reported reverse results. Apart from that, a study [34] showed that the correlation coefficients between cadmium and NAG were higher than those between cadmium and *β*_*2*_-MG, and NAG can be used as the most sensitive marker of tubular dysfunction for monitoring residents in non-populated areas. Moriguchi et al. [35] found similar results that NAG showed the highest correlation with Cd, followed by *α1*-MG, *β2*-MG, and RBP. But an 8-year follow-up study conducted in cadmium-polluted areas in China suggested that *β*_*2*_-MG might be more informative than NAG as an indicator for an individual’s future tubular function [36]. And in our study, when the individuals were divided into males and females, the sensitivity of indicators for tubular function was different between the two genders. The BMD for NAG was lower than that for *β*_*2*_-MG and RBP in male residents, while in females the BMD for NAG and RBP was higher than BMD for *β*_*2*_-MG. Interestingly, previous literatures conducted in Japan or China also showed kinds of gender difference in the BMD values. For example, Kobayashi et al. [27] found that the BMD/BMDL value for NAG was higher than that for *β*_*2*_-MGin both males and females aged ≥50 years, with a 84th or 97.5th percentile as a cut-off. Conversely, another Chinese study [9] indicated that the BMD/BMDL value for NAG was the highest in males, while in females the BMD/BMDL value for*β*_*2*_-MG was higher than other two indicators. In these studies, the BMD was all estimated using the Benchmark Dose Software, which requires categorization of the subjects into several groups. Therefore, the results may fluctuate depending on categorization of the exposures, the number of categories, the dose-intervals, or the cut-off value. The effects of these covariates might contribute to explain the gender difference of BMD values for different renal indicators. We should also pay attention to other tubular effect markers such as glomerular filtration rate (GFR) [37], urinary metallothionein (UMT) [17], and urinary α1-MG, which had also been used as biomarkers of renal dysfunction in some populations. Studies using α1-MG as an effect marker suggested that BMD and BMDL for *α*1-MG had higher sensitivity than that for *β*2-MG [20,38,39]. It is important to select a promising marker of cadmium-induced tubular dysfunction, and more work needs to be done to estimate the BMD in polluted and in non-polluted areas in China.

In the present study, no significant correlation between age and urinary cadmium level was found in non-polluted populations. This is similar to a study conducted by Shimizu et al. [28]. Another study [40] conducted in Thai populations residing in a cadmium-polluted area showed that age was not associated with urinary cadmium levels. Sakuragi et al. [20] carried out a study to determine BMD and BMDL values of populations with no anthropogenic exposure to Cd in Japan, and they also found that age did not show significant correlations with BMD or BMDL for *β*_*2*_-MG and NAG. However, all participants recruited in that Japanese study were over 50 years old, which was different from our study. Although age was associated with the three biomarkers in our study, its influence was relatively weak compared with urinary cadmium. Briefly, these studies indicated that it was suitable not to take age into consideration on calculation of BMDL values.

In our study, the BMD method was used to estimate BMD and BMDL values of Cd in male and female residents in non-polluted areas in China, respectively. The results indicated that it was a suitable method for urinary Cd BMD and BMDL estimation. The strength of the BMD method is the extensive usage of this method in assessing health risk of environmental contaminants, and BMD method is not constrained to one experimental dose, but utilizes the information from the whole dose-response curve to a greater extent and depends on less sample size. The limitations of BMD method lie in that categorization of the subjects could decrease statistical power of the results. This is why a new hybrid approach method has been developed recently for estimating BMD and BMDL [22,37,41]. Little information would be lost and the statistical validity and efficiency is higher using this method because the BMD and BMDL were estimated based on continuous exposure and continuous effect markers, thereby avoiding the categorization of the subjects [42]. However, Wang et al. [29] considered that the results of hybrid approach would be biased due to the variance of the data and other variables, such as population demographics (e.g., race, age, sex, and so on) and Cd exposure levels. What’s more, a recently published meta-analysis [43] showed that the BMD for cadmium was not significantly different between hybrid and conventional BMDs approaches. Generally, we would consider evaluating BMD and BMDL values of Cd using the hybrid approach in our future research, due to the mentioned advantages of hybrid approach in many studies.

In conclusion, the present BMD/BMDL values for tubular effects, using a cutoff of 90% and a BMR of 5% or 10%, were lower and closer to the critical levels from previous studies. Our results showed Cd-induced tubular injury in cadmium non-polluted areas in China. RBP was less sensitive than *β*2-MG or NAG in response to Cd exposure, and *β*2-MG was the most sensitive indicator in women, and NAG was the most sensitive one in men. Although certain population groups especially those affected by diabetes are at increased risk of developing kidney disease and manefesting abnormal nephrotoxicity biomarkers [44,45], this possibility can be excluded because all the people recruted in our study were free from diabetes. What’s more, environmental risk factors including lead, mercury, and occupational solvents are also likely to play a role in the development of kidney disease [8]. Because environmental exposures are preventable, the identification of these risk factors would help people with less kidney disease. Certainly, the sample size of our study is not as large as some previous studies performed in Japan, but our work is still valuable for establishment of BMDL values in China and may contribute to a more comprehensive understanding of the association between nephrotoxicity and cadmium exposure.

## Supporting Information

S1 Table Data set of urinary cadmium, RBP, β2MG, and NAG showing by ug/L and ug/g Crin males and females living in a Cd non-polluted area in China. Cr, creatinine; RBP, retinol binding protein; *β*2-MG, *β*2-microglobulin; NAG, N-acetyl-*b*-glucosaminidase.
(DOCX)
