# Supplementary material for: Application of the Benchmark Dose (BMD) Method to Identify Thresholds of Cadmium-Induced Renal Effects in Non-Polluted Areas in China
Source: PLoS One. 2016 Aug 18;11(8):e0161240. doi: 10.1371/journal.pone.0161240 (PMC4990304; doi:10.1371/journal.pone.0161240)
Supplement: S1 Table — Cr, creatinine; RBP, retinol binding protein; β2-MG, β2-microglobulin; NAG, N-acetyl-b-glucosaminidase. (DOCX) [file pone.0161240.s001.docx]

**S1 Table. Data set of urinary cadmium, RBP, β2MG, and NAG showing by ug/L and ug/g Cr in males and females living in a Cd non-polluted area in China.**

| Sex | Indexes | Mean | SD | Minimum | Maximum |
| --- | --- | --- | --- | --- | --- |
| Male | Creatinine (ug/L) | 150.12 | 81.04 | 5.19 | 496.00 |
|  | Urinary cadmium |  |  |  |  |
|  | ug/L | 1.21 | 1.60 | 0.00 | 16.49 |
|  | ug/g Cr | 0.92 | 1.04 | 0.00 | 8.28 |
|  | Urinary RBP |  |  |  |  |
|  | ug/L | 155.95 | 284.43 | 25.40 | 2318.31 |
|  | ug/g Cr | 101.96 | 128.54 | 13.02 | 927.06 |
|  | Urinary*β*2-MG |  |  |  |  |
|  | mg/L | 0.40 | 0.87 | 0.01 | 9.85 |
|  | ug/g Cr | 0.30 | 0.50 | 0.01 | 5.92 |
|  | Urinary NAG |  |  |  |  |
|  | U/L | 13.25 | 11.88 | 0.00 | 93.37 |
|  | U/g Cr | 9.18 | 6.65 | 0.00 | 50.80 |
| Female | creatinine (ug/L) | 111.37 | 68.56 | 4.56 | 336.00 |
|  | Urinary cadmium |  |  |  |  |
|  | ug/L | 0.95 | 0.88 | 0.00 | 4.89 |
|  | ug/g Cr | 1.07 | 1.06 | 0.00 | 7.85 |
|  | Urinary RBP |  |  |  |  |
|  | ug/L | 87.63 | 124.51 | 26.12 | 1372.12 |
|  | ug/g Cr | 98.22 | 110.40 | 14.03 | 914.59 |
|  | Urinary*β*2-MG |  |  |  |  |
|  | mg/L | 0.23 | 0.49 | 0.01 | 8.50 |
|  | ug/g Cr | 0.30 | 0.60 | 0.00 | 5.92 |
|  | Urinary NAG |  |  |  |  |
|  | U/L | 9.75 | 9.30 | 0.00 | 80.68 |
|  | U/g Cr | 9.14 | 7.21 | 0.00 | 47.87 |
| Total | creatinine (ug/L) | 130.90 | 76.78 | 4.56 | 479.00 |
|  | Urinary cadmium |  |  |  |  |
|  | ug/L | 1.04 | 1.08 | 0.00 | 12.01 |
|  | ug/g Cr | 0.99 | 1.05 | 0.00 | 8.28 |
|  | Urinary RBP |  |  |  |  |
|  | ug/L | 122.06 | 222.64 | 25.40 | 2318.31 |
|  | ug/g Cr | 100.11 | 119.82 | 13.02 | 927.06 |
|  | Urinary*β*2-MG |  |  |  |  |
|  | mg/L | 0.32 | 0.72 | 0.01 | 9.85 |
|  | ug/g Cr | 0.30 | 0.55 | 0.00 | 5.92 |
|  | Urinary NAG |  |  |  |  |
|  | U/L | 11.51 | 10.81 | 0.00 | 93.37 |
|  | U/g Cr | 9.16 | 6.93 | 0.00 | 50.80 |

Cr, creatinine; RBP, retinol binding protein; *β*2-MG, *β*2-microglobulin; NAG, N-acetyl-*b*-glucosaminidase.
